# Beyond BRCA: The Emerging Significance of DNA Damage Response and Personalized Treatment in Pancreatic and Prostate Cancer Patients

**DOI:** 10.3390/ijms23094709

**Published:** 2022-04-24

**Authors:** Bruna Dalmasso, Alberto Puccini, Fabio Catalano, Roberto Borea, Maria Laura Iaia, William Bruno, Giuseppe Fornarini, Stefania Sciallero, Sara Elena Rebuzzi, Paola Ghiorzo

**Affiliations:** 1 IRCCS Ospedale Policlinico San Martino, Genetics of Rare Cancers, 16132 Genoa, Italy; brunasamia.dalmasso@hsanmartino.it (B.D.); william.bruno@unige.it (W.B.); 2 IRCCS Ospedale Policlinico San Martino, Medical Oncology Unit 1, 16132 Genoa, Italy; albertopuccini1@gmail.com (A.P.); catalan.fab@gmail.com (F.C.); roby.borea@gmail.com (R.B.); iaia.mlaura4@gmail.com (M.L.I.); giuseppe.fornarini@hsanmartino.it (G.F.); stefania.sciallero@hsanmartino.it (S.S.); 3 Department of Internal Medicine and Medical Specialties, University of Genoa, 16132 Genoa, Italy; saraelena89@hotmail.it; 4 Ospedale San Paolo, Medical Oncology, 17100 Savona, Italy

**Keywords:** DNA damage response, BRCA, mismatch repair, homologous recombination, genetics, PARP inhibitors, pancreatic cancer, prostate cancer

## Abstract

The *BRCA1/2* germline and/or somatic pathogenic variants (PVs) are key players in the hereditary predisposition and therapeutic response for breast, ovarian and, more recently, pancreatic and prostate cancers. Aberrations in other genes involved in homologous recombination and DNA damage response (DDR) pathways are being investigated as promising targets in ongoing clinical trials. However, DDR genes are not routinely tested worldwide. Due to heterogeneity in cohort selection and dissimilar sequencing approaches across studies, neither the burden of PVs in DDR genes nor the prevalence of PVs in genes in common among pancreatic and prostate cancer can be easily quantified. We aim to contextualize these genes, altered in both pancreatic and prostate cancers, in the DDR process, to summarize their hereditary and somatic burden in different studies and harness their deficiency for cancer treatments in the context of currently ongoing clinical trials. We conclude that the inclusion of DDR genes, other than *BRCA1/2*, shared by both cancers considerably increases the detection rate of potentially actionable variants, which are triplicated in pancreatic and almost doubled in prostate cancer. Thus, DDR alterations are suitable targets for drug development and to improve the outcome in both pancreatic and prostate cancer patients. Importantly, this will increase the detection of germline pathogenic variants, thereby patient referral to genetic counseling.

## 1. Introduction

At each cell division, there is a risk of errors occurring in the DNA replication machinery. DNA replication errors occur more frequently in the presence of DNA damaging agents, both endogenous and exogenous. For instance, reactive oxygen species (ROS) generated during metabolism and the inflammatory process can alter the biochemical structure of nucleic acids. Exogenous factors influencing the likelihood of replication errors include UV rays, which alter the structure of nucleic acids and can lead to the formation of pyrimidine dimers. Moreover, ionizing radiation, such as X-rays and γ-rays, can cause both single-strand and double-strand DNA breaks, whereas exposure to alkylating agents can lead to the formation of DNA adducts and DNA crosslinks [1].

To correctly maintain the fidelity of the genetic code, cells have developed sophisticated methods to sense and repair DNA replication errors. When those errors cannot be repaired, mechanisms are put in place to force the cell to undergo senescence and/or eliminate the cell through apoptosis. However, the disruption of the DNA damage response (DDR), resulting in the escape of death/senescence or uncontrolled cell proliferation with DNA replication errors, leads to genomic instability, one of the hallmarks of cancer [2].

## 2. DNA Damage Response (DDR)

More than 400 proteins and multiple pathways are involved in the regulatory machinery that constitutes the DDR [3]. The main DDR pathways are: base excision repair (BER), nucleotide excision repair (NER), mismatch repair (MMR), homologous recombination (HR), and non-homologous end joining (NHEJ). Although a subset of genes operates within a single pathway, the different DDR pathways are intertwined, and several genes are involved in the correct functioning of multiple pathways [4].

### 2.1. Base Damage and DNA Single-Strand Breaks

Alterations that change or remove a single base, such as UV-induced cytosine deamination, are addressed by DNA glycosylases belonging to the BER pathway [5]. After the altered base is removed by DNA glycosylases, specific endonucleases, such as Ape1, introduce DNA single-strand (ssDNA) breaks, which are then repaired by DNA polymerase β (POLβ) and XRCC1-DNA ligase IIIa, recruited to the ssDNA break site by the poly(ADP-ribose) polymerase inhibitor (PARP) 1 (PARP1) [6].

Another major DNA excision repair is NER, which removes a broad spectrum of single-strand lesions that impair correct DNA coiling [7]. Unlike BER, NER consists of the removal of an oligonucleotide, followed by the repair of the excision using the opposite DNA strand as a template [8].

DNA base–base mismatches and insertion–deletion loops (IDL) can be generated during DNA replication. These types of DNA errors are identified and repaired by the MMR pathway [9]. The main MMR genes frequently altered in cancer are: *MLH1*, *MSH2*, *MSH6*, and *PMS2* genes. The MMR is identified and initiated by the *MSH2*/*MSH6* heterodimer (mutSα), and then completed by another heterodimer (mutLα) formed by *MLH1* and *PMS2* [10].

In addition to the editing of mismatched base pairs, MMR genes can also regulate the HR pathway, in order to maintain the correct functioning of DNA double-strand (dsDNA) break repair [11].

### 2.2. DNA Double-Strand Breaks

dsDNA breaks are the most severe form of DNA damage, resulting in DNA replication arrest if not repaired [12]. HR is a high-fidelity pathway involved in the restoration of dsDNA breaks [13]. In HR, DNA damage is sensed by ATM, which activates several proteins, including BRCA1 and BRCA2, after which DNA ends are resected from 5′ to 3′ by the MRN complex (formed by the proteins RAD50, MRE11, and NBS) [14]. An array of other molecules, including the RAD51 paralogs, invade with the stranded filaments the sister chromatid, which is then used as a template by DNA polymerases to elongate the stranded filaments [15].

An alternative dsDNA repair is carried out through the NHEJ pathway, which does not require an intact template [16]. Briefly, through the interaction of the kinase proteins Ku70 and Ku80, DNA-PKcs, and ATM, and the MRN complex, the stranded ends are cleaved to a lesser extent than that of HR and are then ligated together by specific ligases. Although this mechanism repairs dsDNA lesions, it is more error-prone, as it results in deletions with the consequent loss of genetic information [17].

## 3. Harnessing DDR Deficiency for Cancer Treatment

With the advent of targeted therapies, DDR genes, frequently altered in cancer, have been studied to implement and personalize cancer medical treatment [18].

The rationale of using DDR-targeting agents is to induce cell death through synthetic lethality by blocking a complementary pathway in cells lacking one DDR pathway [19]. Specifically, poly(ADP-ribose) polymerase 1 and 2 (PARP1 and PARP2) enzymes are essential for the normal functioning of BES and its blockade results in the lack of repair of single-strand DNA breaks, with the consequent increased number of errors leading to DNA double-strand breaks that, in the absence of the HR pathway in BRCA1/2 deficient cells, can only be repaired by error-prone mechanisms, such as NHEJ [20]. Moreover, several PARP-inhibitors (PARP-i) also cause an entrapment of PARP at the replication fork, which becomes stalled, and cannot be restarted unless the HR pathway is functioning. There is a growing amount of evidence that this latter mechanism plays a major role in cell death by PARP-i molecules [21,22]

Starting from *BRCA1/2* studies in breast and ovarian cancer [23,24], DDR-targeting drugs are being studied in other neoplasms with a deficiency of *BRCA1/2* or other HR genes, especially pancreatic cancer [25] and castration-resistant prostate cancer [26]. Indeed, clinical data on olaparib, the first PARP-i approved, in pancreatic and prostate cancer were published for the first time in 2014, and even though the size of the study cohort was small (23 pancreatic and 8 prostate), these data led to further clinical investigations [27]. Following the success of PARP-i, novel molecules targeting other DDR genes and pathways are being studied.

## 4. Pancreatic Cancer and Prostate Cancer

Exocrine pancreatic cancer is one of the most lethal malignancies, being the fourth cause of death by cancer considering both sexes together, and predicted to be the second by 2030, with a survival rate at five years from diagnosis lower than 10% [28]. Pancreatic adenocarcinoma, in particular ductal adenocarcinoma, is the most frequent form of pancreatic cancer, constituting more than 85% of all pancreatic cancer cases [29].

Traditional chemotherapy and radiotherapy regimens can hardly overcome the aggressiveness of this disease, and do not guarantee the same response in different treated patients. Therefore, research is ongoing to identify potentially actionable genes and pathways involved in the genesis and progression of this disease, to improve and personalize pancreatic cancer medical treatment [30].

A subset of 5 to 15% of individuals who develop pancreatic cancer are either younger than expected for this malignancy or have a positive family history of pancreatic cancer and/or multi-tumor syndromes [31]. A germline pathogenic variant in a known pancreatic cancer predisposition gene can be found in less than 20% of these patients, depending on selection criteria and genes tested.

The recent literature shows that genes associated with breast and ovarian cancer risk are also the most strongly associated with pancreatic cancer risk, with the exception of *CDKN2A* in some populations [32,33,34]. For example, the DDR genes *BRCA1*, *BRCA2* and *ATM*, or *PALB2*, each one usually found mutated in no more than 3.5% of cases, increase pancreatic cancer risk when altered at the germline level [35]. Based on the available literature, it is estimated that 17 to 25% of pancreatic cancer harbor somatic PVs in one of the genes involved in DDR, mainly those implicated in homologous recombination DNA damage response and repair (HR), such as *BRCA1*, *BRCA2*, *ATM*, *PALB2*, *ATRX*, and *RAD51* [36,37,38,39,40,41,42,43,44].

Prostate cancer is the second most frequent malignancy in males worldwide (the first in western countries and in most African countries), representing 14.3% of all new cancers in males 2020, and the fifth cause of death by cancer in this population [28]. Although the majority of prostate cancers are low risk and/or diagnosed at an early stage, a subset of them displays an aggressive behavior. The initial medical approach to prostate cancer is based on the use of androgen-blocking agents, but a high proportion of metastatic prostate cancers tend to rapidly develop resistance to androgen-blocking agents. Metastatic castration-resistant prostate cancer (mCRPC) patients have a dismal prognosis, as median survival does not exceed two years [45].

The majority of mCRPC samples harbor clinically actionable molecular alterations. With regard to DDR genes, somatic mutations are found in around 23% of mCRPC, and up to 10% of individuals diagnosed with metastatic prostate cancer harbor a germline mutation [46,47]. Of the latter, more than half show loss of heterozygosity in the tumor [48].

In this review, we provide an overview on the DDR genes altered in both pancreatic and prostate cancers. Considering that the mutation rates of DDR genes vary considerably across different studies, and that differences in size cohorts and DNA sequencing methods are likely to be among the reasons of these discrepancies, we only considered original papers, reviews, and systematic reviews/meta-analyses involving at least 200 cases analyzed through multi-gene panel, exome, or genome sequencing, and we set a cut-off of at least 0.2% for reporting mutation rates.

In addition, we summarize the clinical implications of targeting these genes in the context of currently ongoing clinical trials.

## 5. Potentially Actionable DDR Genes in Common between Pancreatic Adenocarcinoma and Castration-Resistant Prostate Cancer (mCRPC)

### 5.1. BRCA1 and BRCA2

Identified in 1994 and 1995 by positional cloning [47], *BRCA1* and *BRCA2* are two of the main genes that control chromosomal stability. Indeed, upon phosphorylation by protein kinases, such as ATM, ATR, and CHK2, BRCA1 and BRCA2 become part of the macromolecular complexes necessary to repair DNA double-strand breaks through HR [49]. *BRCA1*- and *BRCA2*-deficient cells, lacking both copies of either of the two genes, show a high rate of mutations in multiple genes, including gatekeeper genes, such as *CDKN2A*, a phenomenon that can lead to neoplastic degeneration.

Germline biallelic pathogenic variants (PVs) in *BRCA1* or *BRCA2* result in different forms of Fanconi Anemia (FA), a syndrome characterized by short stature, multi-organ malformations, neurodevelopmental disorders, and cancer susceptibility [50]. On the other hand, the inheritance of a single allele with a PV predispose to several types of cancers following a second hit, including pancreatic cancer [51]. *BRCA1* and *BRCA2* PVs can be found in up to nearly 5% of the primary tumors of pancreatic cancer, with the highest frequencies in cohorts enriched for high-risk pancreatic cancer cohorts enriched for familiar cases [52].

In prostate cancer, *BRCA1*/2 mutations represent around 13% of the of DDR genes alterations in tumor samples and 51% of all germline variants found in individuals affected by prostate cancer, with *BRCA2* harboring the majority of variants [46].

Germline *BRCA1* and *BRCA2* PVs increase the developing prostate cancer with a higher likelihood of aggressive disease for *BRCA2* PV carriers [53,54,55,56]. Overall, *BRCA1/2* PVs can be found in both germline and somatic samples from pancreatic cancer patients at similar rates. Conversely, *BRCA1/2* PVs in prostate cancer occur more frequently as a somatic hit.

### 5.2. ATM

Ataxia–Telangiectasia mutated (*ATM*) is a large (351KD) PI3/Pi4 kinase with pleiotropic functions. In addition to playing a key role in HR by activating *BRCA1* and *BRCA2*, *ATM* is involved in DNA double-strand break repair via NHEJ. Moreover, ATM is essential for the correct maturation of lymphocytes [57] and the central nervous system [58]. Indeed, the carriers of biallelic *ATM* PVs are affected by ataxia–telangiectasia, a rare syndrome characterized by progressive cerebellar ataxia, skin telangiectasias, an increased susceptibility to hematologic and solid tumors, and immunodeficiency. Heterozygous carriers of *ATM* PVs are at increased risk of several types of cancer, including pancreatic cancer, and it is estimated that up to 3% of high-risk individuals who develop pancreatic cancer harbor an *ATM* PV [25,35,59,60].

*ATM* has also been proposed as a prostate cancer predisposition gene and has been found altered at the germline level in both prostate cancer patients with suspected familial cancer syndromes and in apparently sporadic prostate cancer patients [48,61,62]

Somatic ATM loss rate can also happen in sporadic pancreatic cancer and prostate cancer [25,36,37,61,63,64]. Indeed, results from a large pan-cancer WGS study include *ATM* among the top 26 driver genes in these cancers, being found altered in 9 and 7% of pancreatic cancer and prostate cancer samples, respectively [63].

### 5.3. ATR

The Ataxia–Telangiectasia and Rad3-Related Protein (*ATR*) encodes for a serine-threonine kinase that acts as a DNA stress sensor. Specifically, in the presence of DNA ionizing and UV radiation and other genotoxic stressors, as well as in the case of the stalling of the replication fork, ATR activates checkpoint inhibitors, such as CHK1, to arrest cell cycle. Moreover, in cells lacking ATR, the inhibition of ATM or proteins in the ATM pathway is synthetically lethal [65]. The rates of *ATR* PVs, higher in at least 0.2% of the analyzed samples, have been found only as a germline event in pancreatic cancer and prostate cancer patients [35,48].

### 5.4. BRIP1

*BRCA1*-interacting protein (BRIP1) is a helicase that interacts with BRCA1 and promotes its DNA repair activity. It is also known as FANCJ, as it is part of the FA complex J [66]. Similar to *BRCA1/2*, biallelic germline PVs in the *BRIP1* gene are found in children with FA syndrome. Germline monoallelic PVs increase the risk of cancer and are found in up to 1% of pancreatic cancer patients. [35,60]. A similar rate can also be found in sporadic pancreatic cancer samples [36,37]. In prostate cancer, *BRIP1* PVs are less frequent, and rates higher that 0.2% are exclusively of germline origin [61,62].

### 5.5. CHEK1 and CHEK2

Checkpoint Kinase 1 (*CHEK1*) and Checkpoint Kinase 2 (*CHEK2*) encode for two serine-threonine kinases (CHK1 and CHK2, respectively) that are effector kinases acting downstream of ATM and ATR and are involved in cell cycle arrest following DNA damage [67].

ssDNA breaks as well as ssDNA generated following DSB resection in dsDNA break repair or during stalled replication fork ultimately result in the activation of the ATR/ATRIP complex, with subsequent CHK1 phosphorylation [68]. CHK2, on the other hand, is activated through phosphorylation by ATM following dsDNA breaks [67]. Both proteins activate signaling networks, leading to cell cycle arrest [67].

Germline PVs in *CHEK2* increase the risk of breast cancer, albeit the penetrance has not yet been defined [69]. Moreover, germline PVs in both genes have been found in several other cancers, but their role those cancers is still under investigation [70,71,72,73].

PVs in one or both of those genes are more frequent at the germline level, both in pancreatic cancer and prostate cancer. In the latter, germline *CHEK1*/*CHEK2* PVs have been found in up to 4.1% of affected individuals [48,61].

### 5.6. FANCA

The FA Complementation Group A (*FANCA*) gene is the main causative gene of FA, being altered at the germline levels in at least 60% of the affected children [74]. FANCA is part of the FA core complex, a macromolecular structure with ubiquitin-ligase functions belonging to the FA pathway, involved in DNA crosslinks repair and signaling upon replication stress, and acts in close interaction with *BRCA1/2* and *RAD51* to protect the replication fork from stalling [75,76].

FANCA has been associated with pancreatic cancer, being among those genes with germline and PV rates higher than 1% [35]. At the somatic level, *FANCA* has been found mutated in both cancers, with a higher prevalence of PVs in prostate cancer.

### 5.7. Mismatch Repair (MMR) Genes

The mismatch genes are *MLH1*, *MSH2*, *MSH6*, and *PMS2*. Germline PVs in MMR genes predispose to Lynch syndrome, which is characterized by a higher risk of developing non-polyposis-associated colorectal cancer (HNPCC) as well as extracolonic neoplasms, including pancreatic cancer and prostate cancer [77], whereas microsatellite instability (MSI) due to somatic impairment of MMR can be found in sporadic colorectal cancer. Recently, MMR genes have been implicated in the development of pancreatic and prostate cancers. Indeed, individuals with Lynch syndrome have a higher risk of developing both pancreatic and prostate cancers [78,79].

Germline PVs in MMR genes have been described in both pancreatic cancer and mCRPC unselected for family history [35,60]. MMR PVs are also present in 0.8% of pancreatic cancer samples and in up to 3% of prostate cancer samples [36,61].

### 5.8. NBN

The Nijmegen Breakage Syndrome 1 gene, also called Nibrin (*NBN*), is part of the NBN-MRE11-RAD50 complex. Upon activation by dsDNA breaks, the MRN complex participates in both HR and NHEJ [80]. The biallelic absence of *NBN* characterizes the Nijmegen Breakage Syndrome, a recessive disorder that includes intrauterine growth restriction, microcephaly, increased susceptibility to upper and lower airway infections, and several types of cancer [81,82].

*NBN* germline PVs range from 0.21% in unselected pancreatic cancer patients to 0.59% in familial pancreatic cancer patients [35], as well as in 2% of prostate cancer patients unselected for family history [48]. Conversely, NBN somatic variants are not found at rates higher than 2% in either of the two cancers.

### 5.9. PALB2

Partner and Localizer of *BRCA2* (*PALB2*), also known as *FANCN*, is a moderate-risk breast cancer susceptibility gene involved in both to the FA pathway. Moreover, PALB2 binds BRCA1 and BRCA2, forming a complex necessary for HR [83]. Biallelic loss-of-function *PALB2* variants cause a form of Fanconi anemia. *PALB2* somatic PV occur in both pancreatic cancer and prostate cancer samples, whereas germline PV have been described in pancreatic cancer patients, at a slightly higher rate in high-risk compared to apparently sporadic patients (0.97% and 0.1–0.65%, respectively) [35,59].

### 5.10. RAD51 Paralogs

The *RAD51* paralogs are: *RAD51B*, *RAD51C*, *RAD51D*, *XRCC2*, and *XRCC3*. Each of these proteins work in an intertwined way with the others, forming macromolecule complexes essential for HR.

Considering that cells with null *RAD51* paralog genes show deficient HR, tumors that lack at least one of those genes could be considered targets for therapy with PARP-I and other drugs directed at HR. Indeed, human cell lines deficient for *RAD51* paralogs, with the exception of *RAD51B*, show marked genomic instability and sensitivity to both mytomicin C and olaparib [84].

Of all *RAD51* paralogs, the germline *RAD51C* and *RAD51D* variants can be found in both pancreatic cancer and prostate cancer, at a rate lower than 0.5%. Only in prostate cancer, however, *RAD51D* is altered in up to 4% of somatic samples.

### 5.11. The Burden of DDR Deficiency in Pancreatic and Prostate Adenocarcinomas

Overall, genomic aberrations in 11 non-*BRCA* DDR genes are shared by pancreatic cancer and prostate cancer. PVs in those genes are found in up to 16% of germline pancreatic cancer samples. The addition of non-*BRCA* DDR genes triplicates the mutational burden given by *BRCA1* and *BRCA2* alone. When looking at somatic PVs, the scenario is comparable, as the highest rate of PVs found in the literature increases from 4.8% to 18.5% when adding other DDR genes to *BRCA1/2*. As for prostate cancer, the addition of the genes included in this review to *BRCA1* and *BRCA2* more than doubles the mutational burden in both germline and in somatic sample PVs (8.6% to 20.7% and 15.2% to 36.9%).

Overall, PVs in both *BRCA1/2* and in non-*BRCA* genes grouped together are slightly more frequent at the somatic level in pancreatic cancer, whereas in prostate cancer, somatic PVs are twice as many as germline PVs.

**Figure 1:**
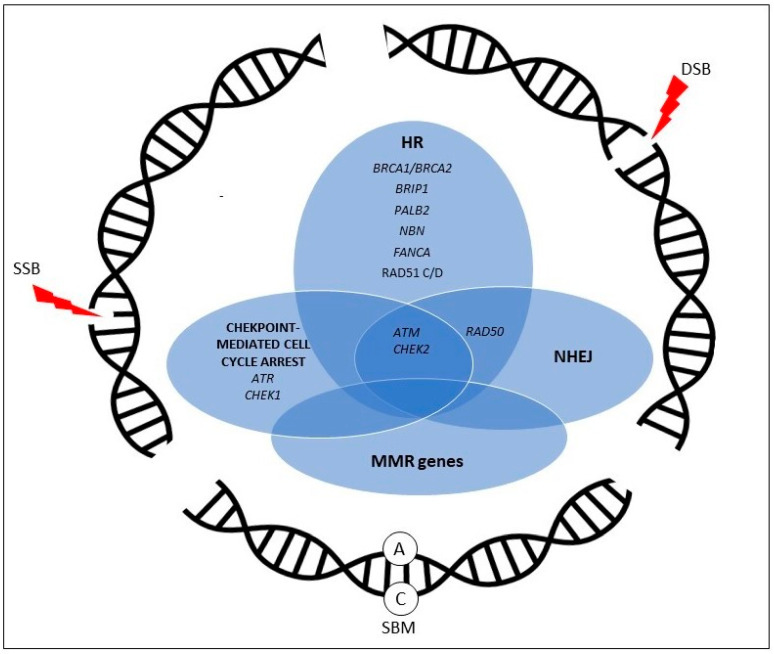
Overview of DNA damage response pathways with genes altered in both pancreatic and prostate cancer. HR = homologous recombination, NHEJ = nonhomologous end-joining, MMR = mismatch repair, SBM = single-base mismatch.

In fact, there is a striking difference between the two types of tumors in what concerns the rate of somatic PVs, which are almost doubled in prostate cancer compared to pancreatic cancer (36.94% vs. 18.5%). A detailed overview of the mutation rates of the above-mentioned 11 genes is shown in Table 1, and their role in DDR pathways is summarized in Figure 1.

## 6. DDR and Cancer Treatment in Pancreatic Adenocarcinomas

### 6.1. DDR Pathogenic Variants in Pancreatic Cancer

DDR gene alterations are correlated with increased overall survival (OS) (17.9 versus 9.6 months, *p* = 0.03) compared to patients without a DDR gene alteration [85]. Indeed, in a recent article, the median overall survival (mOS) in patients with *ATM* alterations was 40.2 months compared with 15.5 months in the control population (hazard ratio (HR) = 0.14, 95% confidence interval (CI) = 0.04 to 0.47, 2-sided *p* = 0.001). These findings suggest that pathogenic ATM alterations may be prognostic for improved outcomes in patients with pancreatic cancer [86]. Interestingly, DDR mutations seem to correlate with a significantly longer OS in patients treated with 5-Fluorouracile, irinotecan, and oxaliplatin (FOLFIRINOX) compared to patients without mutations in DDR genes [87]. The success of novel target therapies with PARP1-i in pancreatic cancer has prompted researchers to explore to a greater extent the role of DDR in pancreatic cancer genesis and progression, in order to broaden the set of patients who could benefit from those therapies and also identify potential targets for the development of other targeted therapies focused on DDR deficiency.

In this review, we report the different mutations in DDR pathways currently investigated in pancreatic cancer patients as promising targetable alterations.

### 6.2. Clinical Trials Results in Pancreatic Cancer

Ongoing clinical trials in pancreatic cancer are reported in Table 2.

The POLO study is a phase III clinical trial conducted on *BRCA1/2* mutated metastatic pancreatic cancer patients treated with olaparib vs. placebo as a maintenance therapy after a platinum-based chemotherapy. In this study, 3315 patients were screened, 154 underwent a 3:2 ratio randomization, and 92 received olaparib. The progression free survival (PFS) was longer in the olaparib group (7.4 vs. 3.8 months; HR = 0.53) [88]. Results from the POLO trial led to olaparib being approved by the US Food and Drug Administration (FDA) and the European Medicines Agency (EMA) in pancreatic cancer with germline *BRCA1/2* PV, as maintenance after a platinum-based chemotherapy. Updated results in 2021 showed that mOS was similar between olaparib and placebo, although this is probably due to 29% of the crossover of patients from placebo to PARP inhibitor upon progression. More importantly, the OS rate at 36 months was 33.9% for olaparib and 17.8% for placebo, which are impressive results considering the poor prognosis of pancreatic cancer patients [89].

Another PARP-i that has been investigated in pancreatic cancer patients harboring DDR gene mutations is Veliparib. In a phase I trial, Veliparib, in association with gemcitabine and cisplatin as first-line treatment in both *BRCA1/2* germline mutated and wild-type patients, showed a good safety profile, although the clinical response was reported exclusively in the mutated group [90]. On the other hand, a phase II trial involving *BRCA1*/2 or *PALB2* mutated pancreatic cancer patients did not demonstrate a benefit in response rate adding veliparib to Cisplatin + Gemcitabine treatment [91]. However, this trial established cisplatin + gemcitabine as a possible new standard treatment in patients harboring *BRCA1*/2 or *PALB2* mutation [91].

Moreover, a phase II trial tested Veliparib in previously treated *BRCA1/2* mutated pancreatic cancer patients. The trial did not show an objective response rate (ORR), although 25% of patients were stable for 4 months [92]

An ongoing clinical phase II clinical trial (NCT02890355) is testing 5-fluorouracile + irinotecan (FOLFIRI) + veliparib as second-line treatment in pancreatic cancer patients with or without *BRCA1/2* mutation. Preliminary results showed no difference in OS between the FOLFIRI group and FOLFIRI + veliparib [93].

In a phase I/II trial, veliparib in association with 5-fluorouracile and oxaliplatin showed promising results in terms of ORR in patients harboring a DDR mutation with a good safety profile [94]. Moreover, veliparib in combination with gemcitabine and radiotherapy demonstrated high tolerability and better mOSin in patients with a DDR pathway alteration compared to patients without DDR mutations [95].

Talazoparib is a new promising PARP-i. In vitro experiments demonstrated that talazoparib selectively targets tumor cells with *BRCA1/2* or *PTEN* mutations with 20- to more than 200-fold greater efficacy than the old generation of PARP-i [96]. A phase I clinical trial of talazoparib in different *BRCA1/2*-mutated tumors showed a good safety profile and promising antitumor activity [97]. Phase II clinical trials in solid tumors are currently ongoing (see Table 2).

The phase II RUCAPANC trial enrolled pancreatic cancer patients with a *BRCA1/2* PV (either germline or somatic) to receive rucaparib. The disease control rate was 31.6% (6 out of 19 patients); hence, the insufficient response rate prompted the closure of the study [98].

Recently, Pishivain et al. published data on 1028 pancreatic cancer patients, 189 of whom harbored an actionable mutation. Of these, 46 (24%) received a molecularly matched therapy [99]. The most common pathway mutated was the DDR (94 of 189 patients). In a subgroup analysis on patients harboring a DDR mutation, 27 received a matched therapy and 67 received an unmatched one. In the subgroup treated with target therapy (PARP-i or ATR inhibitor), the mOS was significantly longer. Similarly, the mOS in patients with an actionable non-DDR mutation was longer in the group treated with the matched therapy [99]. This is a key study to understand the clinical role of using matched therapy in mutated pancreatic cancer patients. According to this study, harboring an actionable alteration and receiving a molecularly matched therapy can predict treatment response and improve mOS compared to receiving a non-matched therapy.

An interesting aspect is the use of HRDness inducers, which can create artificial vulnerabilities allowing the use of PARP-i in patients without BRCA mutation, thus improving the number of patients who can benefit from this therapy [100].

Several ongoing trials are exploring the use of PARP inhibitors in PDAC patients, both as a monotherapy and in combination with other treatments [101,102].

### 6.3. Beyond BRCA

Two phase II ongoing trials are evaluating olaparib in patients with a negative *BRCA* germline mutation, a tumor with a BRCAness phenotype, and a family history of *BRCA*-related cancers, as a second or further line of therapy. Both studies (NCT02677038, NCT02511223) have shown encouraging results, although caution must be taken due to the small sample size (21 and 11 patients); hence, further studies are required [103].

The ataxia–telangiectasia mutated (*ATM*) gene plays an important role in the DDR. Preclinical data in ATM-deficient mouse model showed efficacy of PARP-i and *ATM*-inhibitors (ATM-i) [104]. Different ATM-i have been developed in recent years: KU55933 and KU60019 have shown to be potent radiosensitizers, while AZ31 improved the efficacy of irinotecan therapy [105]. The aToM study is a phase I trial evaluating the safety and efficacy of the ATM-i AZD0156 at increasing doses alone or in combination with other anti-cancer treatments (olaparib or FOLFIRI schedule) in patients with advanced cancers, including pancreatic cancer. Preclinical data demonstrate that the combination of ATM-i with PARP-i enhances the activity of the latter improving DNA DSB and then cell apoptosis [106].

In two phase 2 nonrandomized clinical trials, olaparib was well tolerated and showed limited antitumor activity in patients with advanced, platinum-sensitive pancreatic cancer with alterations in DDR genes, including *ATM* [107].

Preclinical data showed the high activity of ATR-inhibitors (ATR-i) in tumors with a somatic mutation of the ATM pathway, since the ATM-deficient cells rely on the ATR pathway for survival [108,109]. Currently, there are no clinical data available on ATR-i, but several clinical trials are ongoing (see Table 2).

As reported in the previous chapters, checkpoint kinase 1 and 2 (CHK1/CHK2) are activated by ATR and ATM in response to DNA damage or stress [110]. A preclinical study on a CHK1 inhibitor in association with chemotherapy (gemcitabine) and radiotherapy demonstrated a synergic role in killing pancreatic tumor cells [111]. Furthermore, a CHK2 inhibitor has been tested in association with gemcitabine, demonstrating an increased apoptosis of pancreatic tumor cells [111]. No clinical data are available to date, yet CHK inhibitors are being investigated in some clinical trials (see Table 2).

PALB2 has a key role in orchestrating DNA repair, and it is strongly linked to the BRCA1/2, ATM, and ATR pathways [112]. Currently, some trials are evaluating the prognostic role of PALB2 in PARPi-treated patients with mutations in this gene (see Table 2). Nowadays, pancreatic cancer remains a tumor with a poor prognosis and the search for actionable mutations, in order to improve the outcome, is a very hot topic in both preclinical and clinical research.

However, although encouraging results are available in the preclinical setting, clinical data are needed to confirm and validate them.

In the past decade, several treatment strategies have been approved for mCRPC patients and, recently, a better understanding of the underlying biology of prostate cancer allowed researchers to identify and investigate novel therapeutic agents.

The DDR pathways are one of the main actionable molecular alterations of mCRPC, and the investigation of PARP-i has opened a new prospective in the advanced setting of prostate cancer [113].

## 7. DDR and Cancer Treatment in Prostate Adenocarcinomas

Prostate cancer is strongly driven by androgen receptors (ARs) at the beginning of the tumor natural history (“castration sensitive”), while the “castration-resistant” phase of prostate cancer is characterized by tumor heterogeneity caused by the onset of genomic and transcriptomic alterations [113].

HR genes (mainly *BRCA1*, *BRCA2*, and *ATM*), are present in up to 20% of mCRPC patients, at either germline or somatic levels [114].

Testing DDR gene mutations in prostate cancer is clinically relevant, due to their prognostic and predictive values [115]. In prostate cancer patients, harboring a DDR gene mutation, especially a *BRCA2* mutation, is associated with a worse prognosis and a higher Gleason score and stage at diagnosis, as well as with an increased risk of developing distant metastases [116,117].

Moreover, DDR mutations were shown to be positive predictive markers of sensitivity to the platinum-based chemotherapy regimen and PARP-i response in different tumors, including breast, ovarian, and prostate cancers [118].

Due to the promising results of the use of PARP-i in ovarian or breast cancer patients harboring *BRCA1*/2 mutations, several studies have investigated the efficacy of PARP-i in DDR-mutated mCRPC patients, leading to FDA approval for some of these molecules (Table 3) [119,120].

In this review, we report the main clinical trials on the use of PARP-i in mCRPC.

### 7.1. Clinical Trials Results in Prostate Cancer

Several phase 2/3 trials have investigated the efficacy and safety of PARP-i in mCRPC patients (Table 3).

The PROFOUND phase 3 trial assessed olaparib compared with AR-directed therapy (enzalutamide or abiraterone acetate) in mCRPC patients with multiple loss-of-function DDR alterations who progressed to the new hormonal agents [121]. Cohort A included 245 patients with at least one mutation in *BRCA1, BRCA2*, or *ATM*, while cohort B included 142 patients who had a mutation in any of other 12 DDR genes. The somatic mutation status was evaluated with a tissue gene panel analysis. The primary endpoint was radiological PFS (rPFS) in cohort A and secondary endpoints included PFS in cohort B and ORR and OS in cohort A.

The analysis revealed that in cohort A olaparib significantly improved rPFS (7.4 vs. 3.6 months, hazard ratio of 0.34; 95% CI: 0.25–0.47, *p* < 0.0001), the OS of the interim analysis (18.5 vs. 15.1 months, hazard ratio of 0.64; 95% CI: 0.43–0.97, *p* = 0.02), and the objective response rate (ORR) (33% vs. 2%) compared to the hormonal therapy arm. Moreover, olaparib also improved rPFS, OS, and ORR in the overall population (cohorts A and B). This study confirmed the survival improvement and the clinical benefits of olaparib in mCRPC patients harboring DDR alterations opening the path for a new promising class. On the basis of these results, in May 2020, olaparib was approved by the FDA for germline or somatic HR gene-mutated mCRPC patients who progressed to enzalutamide or abiraterone.

The results of the final OS analysis have been subsequently reported [122], confirming the significantly longer OS in patients treated with olaparib in cohort A (19.1 vs. 14.7 months, hazard ratio of 0.69; 95% CI: 0.50–0.97; *p* = 0.02) than in cohort B (14.1 vs. 11.5 months) and overall population (17.3 vs. 14.0 months), despite the substantial crossover from the control therapy to olaparib (66%). Moreover, a sensitivity analysis adjusted for crossover to olaparib showed hazard ratios for death of 0.42 in cohort A, 0.83 in cohort B, and 0.55 in the overall population.

The phase 2 single-arm TRITON2 trial investigated rucaparib 600 mg twice daily in patients with mCRPC with germline or somatic HR gene alterations, detected on blood and/or tumor biopsy. Patients had to be progressed after one or two lines of next-generation AR-directed therapy and one taxane-based chemotherapy. The primary endpoint was ORR and PSA response rate (decrease ≥ 50% from baseline) and secondary endpoints included rPFS and OS.

The study demonstrated the efficacy of rucaparib in 115 mCRPC patients with a *BRCA* alteration, reporting confirmed ORRs per independent radiology review (IRR) and investigator assessment (IA) of 43.5% and 50.8%, respectively, and a PSA response rate of 54.8% [123]. No difference in ORR was seen in patients with a germline versus somatic *BRCA* alteration and in patients with a *BRCA1* versus *BRCA2* mutation, while a higher PSA response rate was observed *BRCA2* mutation. (59.8% vs. 15.4%). Promising survival results were also reported: the median rPFS was 9.0 months per IRR assessment and 8.5 months per investigator assessment. Additionally, although OS data were not mature at the time of the analysis, the 12-month OS reported was 73.0%.

A subgroup analysis revealed that non-BRCA DDR gene alterations, including ATM, CDK12, or CHEK2 mutations, were associated with limited radiographic/PSA responses to rucaparib, while promising responses were observed with mutations in genes that directly interact with the BRCA complex (e.g., *PALB2, BRIP1, FANCA*, and *RAD51B*) [124].

On the basis of this study, in May 2020, rucaparib was approved by the FDA for germline or somatic *BRCA*-mutated mCRPC patients treated with AR-directed therapy and a taxane-based chemotherapy.

In addition to olaparib and rucaparib, other PARP-i have shown promising efficacy results and could be the next FDA-approved PARP-i.

The phase 2 TALAPRO-1 evaluated the efficacy and safety of talazoparib in 104 mCRCP patients with DRR gene defects and measurable disease who received one or two taxane-based chemotherapy regimens for metastatic disease and AR-directed therapy (enzalutamide or/and abiraterone), for metastatic castration-resistant prostate cancers [125]. The study showed that talazoparib was associated with an ORR (primary endpoint) of 30% in the overall population, with a greater antitumor activity in *BRCA1/2*-mutated patients than in those with *PALB2* and *ATM* mutations (46% vs. 25% vs. 12%, respectively). Additionally, the rPFS was higher in *BRCA1*/2-mutated patients compared to the other DDR alterations (overall population: 5.6 months; *BRCA1*/2-mutated patients: 11.2 months; *PALB2*-mutated patients: 5.6 months; *ATM*-mutated patients: 3.5 months). The composite response rate (CRR) (ORR and/or ≥50% PSA decline and/or circulating tumor cell conversion) was 51% in the overall population and was similar in *BRCA1/2*-mutated and *PALPB2*-mutated patients, but lower in *ATM*-mutated patients (72%, 75%, and 24%, respectively).

These results showed that talazoparib has encouraging antitumor activity in heavily pretreated mCRPC patients with DDR–HRR gene alterations, especially those with *BRCA1*/2 mutations.

The phase 2 GALAHAD trial investigated the efficacy and safety of niraparib (300 mg daily) in mCRPC patients with DDR defects who progressed on AR-directed therapy and taxane-based chemotherapy [126]. The primary endpoint was ORR and secondary endpoints included PSA response, CRR, rPFS, and OS. In the final study analysis, 289 patients were included in the overall efficacy analysis population showing an ORR in the measurable *BRCA* cohort (*n* = 76) (primary endpoint) of 34.2%, a median rPFS of 8.08 months, and a mOS of 13.01 months. These results in the *BRCA1/2*-mutated patients were better than those observed in the measurable non-*BRCA* cohort (ORR 10.6%, median rPFS 3.71 months and mOS 9.63 months). Additionally, this study concluded that niraparib has encouraging antitumor activity in heavily pretreated mCRPC patients with DDR–HRR gene alterations, especially those with *BRCA1*/2 mutations.

At the ESMO Congress 2021, the biomarker analysis of cohort A of the phase 1b/2 KEYNOTE-365 trial on the combination of pembrolizumab + olaparib in molecularly unselected, docetaxel-pretreated mCRPC patients was presented [127]. The primary endpoints (PSA response and ORR) were higher in *BRCA*-mutated patients compared with patients without a *BRCA* mutation (50% vs. 14% and 33% vs. 6%, respectively) and in patients with an *HR* mutation compared with those without an *HR* mutation (22% vs. 13% and 8% vs. 3%, respectively). According to these results, the combination of pembrolizumab + olaparib have shown promising activity results, regardless of HR mutation status, even though higher response rates were observed in mutated patients.

Recently, two randomized, double-blind phase 3 studies on PARP-i (the PROpel trial and the MAGNITUDE trial) reported the first analyses at the ASCO Genitourinary Cancers Symposium of February 2022 [128,129]

The PROpel trial investigated the combination of abiraterone acetate plus olaparib versus abiraterone acetate + placebo as a first-line therapy of mCRPC patients [129]. The treatment with abiraterone acetate + olaparib significantly prolonged rPFS (primary endpoint) irrespective of the HRR status (24.8 vs. 16.6 months; hazard ratio of 0.66, 95% CI: 0.54–0.81; *p* < 0.0001). OS is currently immature, but a trend in OS favoring abiraterone acetate plus olaparib was observed (HR 0.86). The secondary endpoints of ORR (58.4% vs. 48.1%), time to first subsequent treatment (hazard ratio of 0.74), and time to second PFS (hazard ratio of 0.69) were supportive of activity and long-term benefits.

The MAGNITUDE trial analyzed the combination of abiraterone acetate plus niraparib versus abiraterone acetate plus placebo as a first-line therapy in mCRPC patients with and without HR [128]. Niraparib + abiraterone acetate did not show any benefit in terms of rPFS or biochemical PFS, and for this reason the accrual in this cohort of patients was interrupted. The combination of niraparib + abiraterone acetate significantly improved rPFS (primary endpoint) in the *BRCA1/2* subgroup (hazard ratio of 0.53, 16.6 vs. 10.9 months) and in all HR+ patients (hazard ratio of 0.73, 16.5 vs. 13.7 months). The first interim analysis of OS is immature, but a trend in OS favoring abiraterone acetate + niraparib was observed (hazard ratio of 0.77). The advantage was also observed in terms of ORR, time to subsequent chemotherapy, and time to symptomatic and biochemical progression, in both *BRCA1/2*-mutated and HR+ patients.

### 7.2. Ongoing Clinical Trials

PARP-i represents a novel treatment option for mCRPC patients harboring HR mutations who progressed to chemotherapy and/or next generation AR-targeted therapy.

However, primary resistance to PARP-i may be present upfront and acquired resistance to them may occur, mainly related to the restoration of the HR mechanism. For this reason, ongoing clinical trials are currently investigating PARP-i in different treatment settings and in combination with different types of oncological therapies, including AR-direct therapy, immunotherapy, and tyrosine kinase inhibitor (TKI) [114,130] (Table 4).

Moreover, some clinical trials included as inclusion criteria the presence or absence of specific HR mutations.

#### 7.2.1. PARP-i in Different Treatment Settings

PARP-i are currently investigated in the early stages of prostate cancer as, e.g., neoadjuvant treatment for locally advanced prostate cancer (BrUOG 337 trial—NCT03432897) or high-risk localized prostate cancer (NCT04030559) and in the metastatic hormone sensitive prostate cancer (mHSPC) (TRYUMPH trial—NCT03413995).

#### 7.2.2. PARP-i plus AR-Direct Therapy

Preclinical data have shown synergy between olaparib and drugs targeting the AR-pathway [131,132]. The exact mechanisms are still not completely known, but it seems that novel hormonal agents can induce an HR phenotype in prostate cancer cells, the signaling of AR pathway may be involved in DNA repair, and the *PARP1* gene may be activated in AR transcriptional activity [133].

Therefore, a phase 2 randomized trial was conducted by Clarke et al. to assess the efficacy and tolerability of olaparib in combination with abiraterone compared with placebo plus abiraterone in mCRPC patients previously received docetaxel, irrespective of their HR mutation status [134]. Olaparib in combination with abiraterone provided an additional clinical efficacy benefit compared with abiraterone alone, even though with an increase in serious adverse events.

- Upon on the same rationale, other ongoing phase 3 randomized trials are comparing PARP-i versus placebo in addition to new hormonal agents (e.g., TALAPRO-2 study—NCT03395197 [135]).

#### 7.2.3. PARP-i plus Immunotherapy

PARP-i have shown to have an immunomodulatory effect modulating the tumor immune microenvironment by a wide range of molecular and cellular mechanisms, such as increasing genomic instability, activating immune pathways and increasing PD-L1 expression on cancer cells, which might promote responsiveness to immune checkpoint inhibitors (ICIs) [136].

According to this biological rationale, several studies are investigating the combination of PARP-i and ICIs [137].

The phase I/II study NCT02484404 reported that the combination of olaparib and durvalumab demonstrates efficacy in terms of PSA response (reduction ≥50%) in mCRPC patients who have received prior enzalutamide and/or abiraterone. Patients with DDR mutations exhibited greater survival (in terms of PFS) benefit compared with those without known alterations [138]. Another phase II study is also assessing the efficacy of the combination olaparib plus durvalumab in castration-sensitive biochemically recurrent non-metastatic prostate cancer harboring at least one DDR deleterious mutation (NCT03810105).

The phase 1–2 KEYNOTE-365 study (NCT02861573) investigated the association of pembrolizumab plus olaparib showing antitumor activity in docetaxel-pretreated and molecularly unselected mCRPC patients who previously received less than two second-generation hormone treatments [139].

A phase 3 study (KEYLYNK-010, NCT03834519) is currently ongoing assessing the efficacy and safety of this combination in molecularly unselected mCRPC patients who progressed to taxane chemotherapy and at least one novel hormonal therapy.

Other phase 2 are currently investigating the combination of different PARP-i with ICIs in mCRPC patients (e.g., rucaparib plus nivolumab in the CheckMate 9 KD—NCT03338790).

#### 7.2.4. PARP-i Associated with TKI

Tumor cell growth is influenced by several factors, including the mechanisms of cell repair, which are contrasted by PARP-i, and the signaling of growth factors, which are contrasted by TKI [130]. The double targeting of these patterns may help to treat patients with CRPC.

TKIs include inhibitors of angiogenesis (e.g., cediranib in NCT02893917) or of the AKT pathway (e.g., ipatasertib in NCT03840200].

#### 7.2.5. PARP-i Associated with Other Treatments

Other combination strategies with PARP-i include therapies that induce DNA damage/replication stress enhancing the activity of PARP-i [130]. Other agents target DDR (ATR-i AZD6738 in NCT03787680), radionuclides (Radium-223 dichloride in NCT03076203 and NCT03317392; Lutetium 177 dotatate in NCT03874884), radiotherapy (NCT04037254), and bipolar androgen therapy (alternating between castration and supraphysiologic testosterone in NCT03516812).

### 7.3. Beyond BRCA in Prostate Cancer

As the main clinical trials’ results on PARP-i in mCRPC were obtained in patients with *BRCA1/2* mutations and data for other DDR genes come from subgroup analyses, the efficacy of PARP-i remains unclear in patients harboring non-BRCA DDR mutations. The “BRCAness” status is defined as a HR alteration not due to *BRCA1*/2 mutations, but to other DDR genes, such as *PALB2, ATM*, *ATR*, *CDK12*, *CHEK1*, *FANC*, and *RAD51/54* [140].

#### 7.3.1. PARP-i

The phase 3 PROfound trial reported that olaparib was less effective in terms of ORR and PFS in the cohort B of patients harboring non-BRCA DDR alterations compared to cohort A of *BRCA*-mutated patients [141]. These data were also observed in two phase II trials on olaparib (TOPARP-B) and rucaparib (TRITON2) [120,124].

The phase II TOPARP-B study assessed the association between olaparib and non-BRCA DDR mutations with the aim to extend the validation of olaparib in DDR-mutated mCRPC [120].

In this study, the antitumor activity of olaparib was higher in *BRCA1*/2 patients, but it was also observed in other DDR gene mutations, especially in the *PALB2* and *ATM* subgroups [120].

The ad hoc analysis of the TRITON2 trial on patients with a non-*BRCA* DDR gene alteration, especially *ATM*, *CDK2*, or *CHECK2* mutations, showed a limited ORR and PSA response rate. On the other hand, responses were observed in patients with *PALB2*, *FANCA*, *BRIP1*, and *RAD5B* alterations [124].

These results suggest that PARP-i might have a role as monotherapy or in combination with other therapies also in BRCAness mCRPC patients, although with a lower magnitude of benefit compared to BRCA-mutated patients. Further, ad hoc studies are needed to assess the therapeutic role of PARP-i in BRCAness mCRPC patients.

#### 7.3.2. Other DDR Gene Inhibitors

*ATM* and *ATR* genes are involved in complex DDR pathways [114]. Some evidence has suggested that targeting *ATM/ATR* mutated cancer with both PARP-i and ATR-i/ATM-i may be more efficacious than using PARP-i alone [142]. Several ATR-i and ATM-i are currently being investigated in early clinical trials as monotherapy or in association with PARP-i, as a double blockade of the DDR pathway, immunotherapy, and hormonotherapy chemotherapy [114].

Other promising preclinical data were obtained with compounds targeting other DDR alterations, including *CHK1*, *WEE1*, *CDK12*, and DNA-PKcs inhibitors, which were tested for activity in preclinical prostate cancer models and are currently under investigation in phase 1/2 trials in different solid tumors, including prostate cancer patients, or specifically in prostate cancer patients [114].

## 8. Conclusions

Pancreatic and prostate cancer were the first cancers after breast and ovarian cancer for which the efficacy of PARP inhibitors was evaluated in the presence of *BRCA1/2* mutations. Since then, the presence and actionability of DDR deficiency in these tumors has been investigated. However, due to differences in cohort selection and DNA sequencing approaches, the burden of PVs in non-*BRCA1/2* DDR genes shared by pancreatic and prostate cancers is not completely defined.

The picture that emerges from the examined literature shows that the addition of other DDR genes to *BRCA1/2* markedly increases the burden of actionable variants, even when looking only at point mutations. We conclude that the inclusion of DDR genes other than *BRCA1/2* shared by both cancers considerably increases the detection rate of potentially actionable variants, which are triplicated in pancreatic and almost doubled in prostate cancer. For prostate cancer, this is particularly relevant at the somatic level, where DDR mutations are almost doubled compared to those found in germline samples, and a germline testing-based approach would miss a large amount of DDR-deficient tumors. Considering the growing applications of DDR-targeting agents in cancer therapy, and the importance of timely genetic testing for patient access to treatment, multi-gene panels for pancreatic cancer and prostate cancer that include these genes should be routinely used in the clinical setting for both cancers.

Overall, DDR alterations are suitable targets for drug development and to improve the outcome in both pancreatic and prostate cancer patients. Importantly, this will increase the detection of germline pathogenic variants, thereby patient referral to genetic counseling.

## Figures and Tables

**Table 1:** Frequency of the pathogenic variants in DDR genes shared by pancreatic and prostate cancers.

	Pancreatic Cancer				Prostate Cancer			
	Germline [25,35,59,60]		Somatic [25,36,37,63]		Germline [48,61,62]		Somatic [61,63,64]	
Gene	Range	Max	Range	Max	Range	Max	Range ^1^	Max
***BRCA1/2***	0.9–5	**5**	0.9–4.8	**4.80**	0.3–8.6	**8.60**	0.6–15.2	**15.2**
*ATM*	2–3.09		2.2–9		1.59–2.3		1.9–7.3	
*ATR*	0.2				0.29			
*BRIP1*	0.22–1		0.48–1		0.28–0.45			
*CHEK1-2*	0.3–2.2		0.2–0.6		1.87–4.1		0.9–1.9	
*FANCA*	1.04		0.6				3	
*MMR*	0.39–1.2		0.8		0.14–1.7		3–6	
*NBN*	0.2–0.59				0.29–2			
*PALB2*	0.1–0.97		0.2–1.2		0.45–0.56		0.4–2	
*RAD50*	0.3–0.36		0.5				1	
*RAD51*	0.35 **^2^**				0.14–0.57 **^2,3^**		0.54 **^2,3^**	
**Non-*BRCA1/2***		**11**		**13.7**		**12.07**		**21.74**
**TOTAL**		**16**		**18.5**		**20.67**		**36.94**

Frequencies are reported as percentages. ^1^ mCRPC, ^2^ *RAD51C*, ^3^ *RAD51D*.

**Table 2:** Ongoing clinical trials on PARP-i in pancreatic cancer.

PARP-i	Clinical Trial	Phase	Patient Population	Somatic Mutations/Germline PVs	Treatment Arm(s)
Olaparib	NCT04548752	2	Pancreatic cancer	*BRCA1/2*	Olaparib + pembrolizumab
	NCT04005690	1	Pancreatic cancer	nd	Olaprib + Cobimetinib
	NCT02498613	2	Advanced Solid Tumors	nd	Olaparib + cediranib
	NCT03162627	1	Solid Tumors	nd	Olaparib + selumetinib
	NCT03842228	1	Advanced Solid Tumors	*ARID1A, ATM, ATRX, BARD1, BRCA1, BRCA2, BRIP1, CDK12, CHEK1, CHEK2, FANCA, FANCL, MRE11A, MSH2, PALB2, PARP1, POLD1, PP2R2A, RAD51B, RAD51C, RAD51D, RAD54L, XRCC2, PTEN, PIK3CA*	Olaparib + durvalumab + Copalinsib
	NCT02511223	2	Pancreatic cancer	BRCAness	Olaparib alone
	NCT02677038	2	Pancreatic cancer	Somatic *BRCA* mutation, Fanconi anemia genes, *ATM* or *RAD51* mutations	Olaparib alone
	NCT02576444	2	Solid tumors	*ATM, CHK2, MRN (MRE11/NBS1/RAD50), CDKN2A/B, APOBEC, IDH1/IDH2, TP52, KRAS, PTEN, PIK3CA, AKT, or ARID1A*	Olaparib + AZD1775 OR AZD5363 OR AZD6738
Rucaparib	NCT03140670	2	Pancreatic cancer	*BRCA 1/2* or *PALB2*	Maintenance after platino-based chemo
	NCT03337087	1–2	Pancreatic, colorectal, gastroesophageal, or biliary cancer	*BRCA 1/2* or *PALB2*	Liposomal Irinotecan, Fluorouracil, Leucovorin Calcium, and Rucaparib
	NCT04171700	2	Solid Tumors	*BRCA1, BRCA2, PALB2, RAD51C, RAD51D, BARD1, BRIP1, FANCA, NBN, RAD51* or *RAD51B*.	Rucaparib alone
Veliparib	NCT02890355	2	Pancreatic cancer	nd	FOLFIRI or mFOLFIRI + Veliparib as II Line
	NCT01585805	2	Pancreatic cancer	*BRCA1/2* or *PALB2* Germline PV	Gemcitabine + Cisplatin with or without veliparib or veliparib wlone
	NCT02723864	1	Solid tumors	nd	M6620 (ATR inhibitor) + veliparib + cisplatin
Niraparib	NCT03601923	2	Pancreatic cancer	Germline PVs or somatic mutation of one of these: *BRCA1/2, PALB2, CHECK2* or *ATM*	Niraparib alone
	NCT04409002	2	Pancreatic cancer	nd	Niraparib + Dostarlimab + RT
	NCT03553004	2	Pancreatic cancer	DDR family mutation	Niraparib alone
	NCT04493060	2	Pancreatic cancer	*BRCA1/2* or *PALB2*	Niraparib + Dostarlimab
	NCT03404960	1–2	Pancreatic cancer	nd	Niraparib + Nivolumab or Ipilimumab after platinum-based chemotherapy
	NCT03207347	2	Solid tumors	*ARID1A, ATM, ATR, BACH1 (BRIP1), BAP1, BARD1, BLM, CHEK1, CHEK2, CDK2, CDK4, ERCC, FAM175A, FEN1, IDH1, IDH2, MRE11A, NBN (NBS1), PALB2, POLD1, PRKDC (DNA-PK) PTEN, RAD50, RAD51, RAD52, RAD54, RPA1, SLX4, WRN*, or *XRCC*	Niraparib
	NCT03209401	1	Solid tumors	*ARID1A, ATM, ATRX, MRE11A, NBN, PTEN, RAD50/51/51B, BARD1, BLM, BRCA1, BRCA2, BRIP1, FANCA/C/D2/E/F/G/L, PALB2, WRN, CHEK2, CHEK1, BAP1, FAM175A, SLX4, MLL2* or *XRCC*	Niraparib + carboplatin
Talazoparib	NCT02286687	2	Solid tumors	Somatic *BRCA1* or *BRCA2*; germline *BRCA, ATM, PALB2*, Fanconi Anemia genes, *ARID1A, MER11, RAD50, NBS1, ATR*; amplification of *EMSY*	Talazoparib
	NCT03565991	2	Solid tumors	*ATM* or *BRCA*	Avelumab and talazoparib
Fluzoparib	NCT04300114	3	Pancreatic cancer	Germline *BRCA/PALB2*	Maintenance after platinum
Prexasertib	NCT02873975	2	Solid tumors	*MYC* amplification, Rb loss, *FBXW7* mutation, *BRCA1, BRCA2, PALB2, RAD51C, RAD51D, ATR, ATM, CHK2*, the Fanconi anemia pathway genes, *CCNE1* amplification of 6-fold or greater, or other genomic or somatic mutation in a known HR gene	Prexasertib (CHK inhibitor)
	NCT03057145	1	Solid tumors	nd	Prexasertib + olaparib
BTT-114	NCT02950064	1	Pancreatic, breast, ovarian, or prostate cancer	*BRCA* or other DNA repair mutations, such as *ATM, CHEK2, PALB2*, and *RAD51D*	BTT-114, a novel platino product
ABT-144	NCT01489865	1	Pancreatic cancer	*BRCA1/2* or *PALB2* or *FANC* mutation or family history	ABT-144 + mFOLFOX6
AZD0156	NCT02588105	1	Solid tumors	nd	(ATM/ATR inhibitor) Alone or in combination
M6620 (VX-970)	NCT02595931	1	Solid tumors	nd	M6620 + irinotecan
AZD6738	NCT03682289	2	Renal, urothelial or pancreatic cancer	*ATM* or *ARID1A*	AZD6738 (ATR inhibitor) +/− olaparib
	NCT02223923	1	Solid tumor	nd	AZD6738 (ATR inhibitor) + radiotherapy
	NCT02630199	1	Solid tumors	nd	AZD6738 (ATR inhibitor) + paclitaxel
	NCT03669601	1	Solid tumor	nd	AZD6738 (ATR inhibitor) + Gemcitabine
Ceralasertib	NCT02264678	1–2	Solid tumors	*ATM* and *BRCA* evaluation	Ceralasertib +/− other drugs
BAY1895344	NCT03188965	1	Solid tumors	*ATM* or other DDR defects	BAY1895344 (ATR inhibitor)
	NCT04514497	1	Solid tumors	*ATM* and other DDR defects	BAY1895344 (ATR inhibitor) + irinotecan

**Table 3:** Main clinical trials and results of PARP-i in mCRPC patients with a HR mutation.

PARP-i	Clinical Trial	Phase and Study Type	Patient Population	Treatment Arm	N pts	Results	Status in February 2022
Olaparib	PROFOUND (NCT02987543)	Phase 3, randomized	Progression to ≥1 novel HT ^1^ Cohort A: BRCA1m, BRCA2m, ATMm. Cohort B: other HR.	Olaparib vs. Enzalutamide or Abiraterone acetate + prednisone	Cohort A: 245 Cohort B: 142	Cohort A: Olaparib > Hormonal therapy in PFS = 7.4 vs. 3.6 mo, HR 0.34; *p* < 0.0001 OS = 18.5 vs. 15.1 mo, HR 0.64; *p* = 0.02 ORR = 33% vs. 2% Cohort A + B: Olaparib > Hormonal therapy in PFS = 5.8 vs. 3.5 mo, HR 0.49; *p* < 0.0001 OS = 17.5 vs. 14.3 mo, HR 0.67 ORR = 22% vs. 4%	FDA-approved in May 2020 Active, not recruiting
	KEYNOTE-365 (NCT02861573)	1b-2, single arm	mCRPC (molecularly unselected, docetaxel-pretreated)	Pembrolizumab + Olaparib (Cohort A)	102	BRCA+ vs. BRCA - PSA response: 50% vs. 14% ORR: 33% vs. 6% HR+ vs. HR- PSA response: 22% vs. 13% ORR: 8% vs. 3%	Active, recruiting
	PROpel (NCT03732820)	Phase 3, randomized	mCRPC 1 L treatment after failure of ADT	Olaparib + Abiraterone Acetate	796	rPFS: 24.8 vs. 16.6 months, HR 0.66, *p* < 0.0001 OS: HR 0.86 ORR: 58.4% vs. 48.1%	Active, not recruiting
Rucaparib	TRITON2 (NCT02952534)	Phase 2, single arm	Progression to 1–2 novel HT ^1^ AND 1 taxane-based CT	Rucaparib	115 BRCAm	ORR IRR = 43.5% ORR IA = 50.8% PSA RR = 54.8% m-rPFS IRR = 9.0 mo m-rPFS IA = 8.5 mo 12-mo OS = 73.0%	FDA-approved in May 2020. Completed
Talazoparib	TALAPRO-1 (NCT03148795)	Phase 2, single arm	Progression to ≥1 novel HT AND 1–2 CT regimens (≥1 taxane-based CT)	Talazoparib	86 overall population 46 BRCA1/2m 4 PALB2m 18 ATMm	ORR overall population = 28% ORR BRCA1/2m = 43.9% ORR PALB2m = 33.3% ORR ATMm = 11.8% m-rPFS BRCA1/2m = 9.3 mo m-rPFS PALB2m = 7.4 mo m-rPFS ATMm = 5.5 mo	Active, not recruiting
Niraparib	GALAHAD (NCT02854436)	Phase 2, single arm	Progression to ≥1 novel HT ^1^ AND ≥1 taxane-based CT	Niraparib	46 BRCA 1/2m 35 non-BRCAm	BRCA1/2m vs. non-BRCAm ORR = 41% vs. 9% PSA RR = 50% vs. 3% m-rPFS = 8.2 vs. 5.3 mo mOS = 12.6 vs. 14 mo	Active, not recruiting
	MAGNITUDE (NCT03748641)	Phase 3, randomized	mCRPC 1 L treatment after failure of ADT	Niraparib + Abiraterone Acetate	423 HR patients	BRCA1/2m vs. non-BRCAm rPFS: 16.6 vs. 10.9 mo, HR 0.53 ORR: 52% vs. 31% HR+ vs. HR- rPFS: 16.5 vs. 13.7 mo, HR 0.73 ORR: 60% vs. 28%	Active, not recruiting

HT: hormonal therapy; N: number; pts: patients; BRCA1m: *BRCA1* mutation; BRCA2m: *BRCA2* mutation; ATMm: *ATM* mutation; HR: homologous recombination DNA damage response and repair; CT: chemotherapy; PALB2m: *PALB2* mutation; PFS: progression-free survival; mo: month; HR: hazard ratio; *p*: *p* value; OS: overall survival; ORR: overall response rate; IRR: independent radiology review; IA: investigator assessment; PSA RR: prostate-specific antigen response rate; m-rPFS: median radiological progression-free survival; 12-mo OS: overall survival at 12 months; FDA: Food and Drug Administration. ^1^ Novel hormonal therapy, e.g.; abiraterone acetate and/or enzalutamide.

**Table 4:** Active clinical trials on PARP-i in prostate cancer.

PARP-i	Clinical Trial	Phase	Patient Population	Somatic Mutations/Germline PVs	Treatment Arm(s)
Olaparib	NCT03434158 (IMANOL)	2	mCRPC	*BRCA1, BRCA2, ATM, FANC, CHEK2, MLH1, MSH2, MSH6, PMS2, PALB2, RAD51C, MRE11*	Olaparib
	NCT03012321	2	mCRPC	*BRCA1, BRCA2, ATM* Other DDR mutations	Abiraterone alone OR Olaparib alone OR Abiraterone + Olaparib
	NCT03516812	2	CRPC	MMR deficiency, HR deficiency	Olaparib + Testosterone
	NCT03317392	1–2	mCRPC	Not required	Olaparib + Radium 233
	NCT03834519 (KEYLYNK-010)	3	mCRPC	Not available	Pembrolizumab + Olaparib Vs Abiraterone OR Enzalutamide
	NCT03432897	2	Locally advanced Prostate cancer	*BRCA1, BRCA 2, ATM, CHEK1, CHEK2,* *FANCONI ANEMIA (FANCL), HDAC2, PALB2, BARD1, BRIP1, CDK12, PPP2R2A, RAD51B, RAD51C, RAD51D*, or *RAD54L*	Olaparib alone
	NCT03810105	2	Castration sensitive AND biochemically recurrent prostate cancer	*BRCA1, BRCA2, ATM, CHEK2, FANCA, RAD51C, RAD51D, PALB2, BRIP1, BARD1*, or *CDK12*	Olaparib + Durvalumab
Niraparib	NCT02854436	2	mCRPC	*BRCA1, BRCA2*, or other DDR alteration	Niraparib alone
	NCT04037254	2	High-risk prostate cancer	Not required	Niraparib + ADT
	NCT04030559	2	High-risk localized prostate cancer	DDR deficiency	Neoadjuvant Niraparib
Rucaparib	NCT03442556	2	mCRPC	DDR deficiency	Rucaparib
	NCT02975934 (TRITON3)	3	mCRPC	*BRCA1, BRCA2* or *ATM*	Rucaparib OR Docetaxel OR Abiraterone OR Enzalutamide
	NCT03413995 (TRIUMPH)	2	mHSPC	*BRCA1, BRCA2, ATM, CHEK2, NBN, RAD50, RAD51C, RAD51D, PALB2, MRE11, FANCA, FANCB, FANCC, FANCD2, FANCE, FANCF, FANCG, FANCI, FANCL, FANCM*	Rucaparib alone
	NCT03533946	2	Non-metastatic prostate cancer	*ATM, ATR, BARD1, BRCA1, BRCA2, BRIP1, CDK12, CHEK1, CHEK2, ERCC3, FAM175A, FANCA, FANCL, GEN1, HDAC2, MLH1, MRE11, NBN, PALB2, PPP2R2A, RAD51, RAD54L*	Rucaparib alone
Talazoparib	NCT03395197 (TALAPRO-2)	3	mCRPC	DDR assessment required	Talazoparib + Enzalutamide
	NCT03330405	2	mCRPC	*ATM, BRCA1* and *BRCA2*	Talazoparib + Avelumab
	NCT04332744	2	mHSPC	Not required	Talazoparib + Enzalutamide

mCRPC: metastatic castration-resistant prostate cancer; mHSPC: metastatic hormone-sensitive prostate cancer; Prostate cancer: prostate cancer; MMR: mismatch repair; HR: homologous recombination; DDR: DNA damage repair; ADT: androgen-deprivation therapy.
